# Medical Society of the County of Erie

**Published:** 1903-11

**Authors:** A. L. Benedict


### Medical Society of the County of Erie.

*Special meeting held October 10, 1903.*

REPORTED BY A. L. BENEDICT, M. D., Acting Secretary.

The president, Dr. ERNEST WENDE in the chair.

Dr. A. L. BENEDICT was appointed secretary pro tem.

Dr. LUCIEN HOWE explained that the object of the meeting was to consider the advisability of memorialising the Medical

Society of the State of New York, in reference to the resolutions passed at the recent meeting of the New York State Medical Association, favoring a union of the two state organisations.

It was moved by Dr. De Lancey Rochester, Dr. Henry Reed Hopkins seconding, that it is the sense of this county society, that the medical profession of the state should be united in one body. Carried.

It was moved by Dr. Hopkins, Dr. Rochester seconding, that a copy of the above resolution be transmitted under the signature of the officers of the meeting and the seal of the society, to the officers of the Medical Society of the State of New York, before its meeting, next Tuesday, October 13, 1903. Carried unanimously.

On motion of Dr. Hopkins, the meeting adjourned.

